# The rise of 3D cellular spheroids: efficient culture via upward growth from a superamphiphobic surface

**DOI:** 10.1093/nsr/nwz158

**Published:** 2019-10-21

**Authors:** Paul J Dyson

**Affiliations:** Institut des Sciences et Ingénierie Chimiques, Ecole Polytechnique Fédérale de Lausanne, Switzerland

Cell culture assays are extensively used in drug discovery and development programs, biomedical research, and in many others areas of science. Currently, 2D monolayer cellular assays serve as the gold standard. However, while they provide seemingly indispensable information on drug activity, it is estimated that of the putative drug candidates selected using these assays, > 90% that progress to *in vivo* experiments immediately fail. Consequently, more physiologically relevant *in vitro* models would considerably reduce the numbers of animals that are required for primary *in vivo* screening, and at the same time would accelerate drug discovery and biomedical research [1]. 3D multicellular spheroids are superior to 2D monolayer cultures, better capturing the natural microenvironment of cells, including the natural gradient of the nutrients, growth and signaling factors, cell heterogeneity, etc. [2], thus bridging the gap between *in vitro* and *in vivo* experiments.

The ability to construct 3D cellular spheroids represents an exciting frontier of biomedical research of relevance to cancer biology, tissue engineering, drug screening and toxicology [3]. Propelled by the many notable advances achieved in culturing 3D cellular spheroids over the past decade, i.e. scaffold-based or scaffold-free techniques such as the hanging drop method, 3D cellular spheroids have become increasingly important in biomedical research. The method selected to generate 3D cellular spheroids depends upon several factors, notably the types of cells under study and the type of information that is expected from the study. Most approaches tend to be labor-intensive and time-consuming; it is particularly challenging to produce uniform spheroids in a reproducible fashion, and some approaches are not well suited to *in situ* imaging, all of which hinders high-throughput screening [4]. Consequently, strategies that overcome one or more of these limitations would be beneficial.

**Figure 1.**
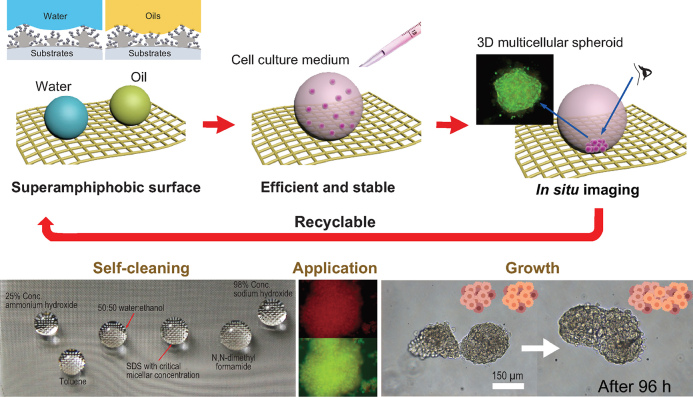
Illustration of the upward growth process of 3D cellular spheroids on a superamphiphobic silica aerogel surface. The antifouling surface prevents cells from adhering to the surface and triggers self-organization of the cells into spheroids. The spheroids can be maintained over prolonged periods facilitating *in situ* imaging. Adapted from [5].

In a research article recently published in NSR, Xu *et al*. disclose an ingenious approach to cultivate 3D multicellular spheroids employing a novel upward culture method that capitalizes on durable superamphiphobic surfaces [5]. To produce 3D cellular spheroids in a bottom-up fashion, Lu's team designed super amphiphobic biointerfaces that combine superhydrophobicity with superoleophobicity. As shown in Fig. 1, the superamphiphobic surface prevents cell–substrate interactions, triggering cell–cell interactions, leading to the efficient formation of 3D cellular spheroids. Notably, the superamphiphobic surface possesses long-term thermal and mechanical stability, which facilitates the prolonged upward growth of 3D cellular spheroids. Such properties are conducive to *in situ* imaging techniques allowing changes in the pathophysiological features of the 3D cellular spheroids to be obtained and providing more complete biological information. As proof-of-concept, size-controlled 3D cellular spheroids were prepared and evaluated in spheroidal fusion and drug screening studies, demonstrating their utility in drug discovery and biomedical research.

In summary, Xu *et al*. have developed a convenient approach for the efficient culture and *in situ* imaging of size-controlled 3D cellular spheroids, which are ideally suited to many applications. Significantly, the approach is highly versatile and can be readily adapted to many areas of translational medicine.
